# Transition zone cells reach G2 phase before initiating elongation in maize root apex

**DOI:** 10.1242/bio.025015

**Published:** 2017-05-11

**Authors:** M. Victoria Alarcón, Julio Salguero

**Affiliations:** 1 Departamento de Hortofruticultura, Centro de Investigaciones Científicas y Tecnológicas de Extremadura, Gobierno de Extremadura, Badajoz 06187, Spain; 2 Departamento de Biología Vegetal, Ecología y Ciencias de la Tierra, Universidad de Extremadura, Badajoz 06007, Spain

**Keywords:** Cell cycle, Maize root apex

## Abstract

Root elongation requires cell divisions in the meristematic zone and cell elongation in the elongation zone. The boundary between dividing and elongating cells is called the transition zone. In the meristem zone, initial cells are continuously dividing, but on the basal side of the meristem cells exit the meristem through the transition zone and enter in the elongation zone, where they stop division and rapidly elongate. Throughout this journey cells are accompanied by changes in cell cycle progression. Flow cytometry analysis showed that meristematic cells are in cycle, but exit when they enter the elongation zone. In addition, the percentage of cells in G2 phase (4C) strongly increased from the meristem to the elongation zone. However, we did not observe remarkable changes in the percentage of cells in cell cycle phases along the entire elongation zone. These results suggest that meristematic cells in maize root apex stop the cell cycle in G2 phase after leaving the meristem.

## Abstract (precis)

**Summary:** Meristematic root cells stop cycling in G2 phase before entering the elongation zone. Remarkable changes in the percentage of cells in the cell cycle phases take place in the transition zone, indicating this zone is a boundary between mitotic and elongation cell processes.

## INTRODUCTION

The maize primary root consists of a cylindrical structure that finishes in a conical shape in the root apex (Alarcón et al., 2014a). According to the processes occurring along its longitudinal axis, the primary root has been dissected in several zones. The root cap is the most external structure in the root tip, and protects the root meristem amongst its other roles. The quiescent centre (QC) is located between the root cap and the meristem, a discoid structure formed of cells which usually do not divide. However, cells above and below the quiescent centre constitute the initial cells of root tissues (Verbelen et al., 2006). The initial cells located below the quiescent centre form the root cap, whereas those located above will form the rest of the root tissues. Cell divisions in different planes, periclinal, anticlinal and tranverse, increase the number of columns that form several root tissues. Most of the cell divisions in the meristem are transversal and resulting cells remain aligned in the same column (Dolan et al., 1993). Longitudinal root growth requires both cell division and elongation. Divisions of initial cells produce two cells: the distal daughter cell close to the meristem, which retains its status of initial cell; and the basal cell, which will elongate parallel to the root axis when it reaches the elongation root zone. Therefore, the size of the meristem does not vary as the number of initial cells does not change. Basal daughter cells initiate the transition from division to elongation, and go into the elongation zone where cell division ceases and cell elongation increases as cells abandon the meristem. The distal elongation zone (DEZ), in which cells resemble meristematic cells, was initially named post-mitotic isodiametric growth zone (Ishikawa and Evans, 1992); cells in this zone elongate at a low rate, and it has been also described as a transition zone (Baluška et al., 2001). As cells reach regions far away from the meristem, the elongation rate increases rapidly and cells then belong to a rapid cell elongation zone (Verbelen et al., 2006). In maize, three major growth regions in the root apex have been distinguished: (a) meristem from root cap junction (RCJ) to the end of the first mm; (b) transition zone from 1.5 to 2.5 mm from RCJ (approximate value); and (c) elongation region from 3.5 to 6 mm (Baluška et al., 2001; Alarcón et al., 2016). Although elongation in maize root extends to 10 mm from RCJ, elongation rate decreases gradually from 6 to 10 mm, where elongation totally stops.

In summary, root elongation requires cell division in the meristem, which provides cells to the elongation zone; in this region, cells grow longitudinally resulting in increased root length. Therefore, root rate elongation is controlled by both cell production and cell elongation. The amount of cells produced in the meristem depends on the number of meristematic cells and the rate of division (Beemster and Baskin, 1998). As the cell cycle determines the number of cells that can be incorporated to the elongation zone, cell cycle rate in the meristem is fundamental to root growth. Cell division has been located by several methods, such as mitotic index, and labelled mitosis after applications of tritiated thymidine, BrdU and EdU (Stroobants et al., 1990; Kotogány et al., 2010; Alarcón et al., 2016). Meristem size has also been determined by measuring the distance between the QC and the first elongated cell (Moubayidin et al., 2013). These works have provided information about the extension of the meristematic zones, but not about which cell cycle phase the cells are in. It is assumed that once cells leave the meristem and reach the transition zone, they exit the cell cycle and elongate (Perilli et al., 2012).

Flow cytometry is a powerful tool in analysing plant cell cycle progression, as it allows us to distribute a determinate cell population in several groups according to the quantity of DNA (Doležel, 1997; Doležel et al., 1992). In a typical DNA profile, nuclei are mostly sorted into two well-defined peaks that correspond to nuclei in G0/G1 and in G2/M phases. These peaks represent DNA content before and after replication; in a linear channel, the cells in G2/M phase have twice as much DNA as in phase G0/G1. Cells in replication are located between the two peaks, and the closer they are to G2/M phase, the closer they are to finishing replication. Frequency of S-phase cells is a basic parameter in cell cycle studies of plants, as it indicates how many cells are in the cell cycle, i.e. if cells are in cycle or they have abandoned the cell cycle (Reichheld et al., 1999).

In this paper, we analysed the cell cycle along the maize primary root in order to know how the cell cycle progresses in several zones of primary root, which have been selected according to the processes that take place in each. For this purpose, we analysed cell distribution along G0/G1, G2/M and S-phases to determine the cell cycle activity when cells are located in a certain root zone. This study allows insight into the changes occurring in the cell cycle when cells leave the meristematic activity to initiate cell elongation in the maize root apex.

## RESULTS AND DISCUSSION

### Cell cycle along the maize primary root

Maize primary root was divided into consecutive segments that indicated the limits of root cap, apical meristem elongation zone and differentiation zone, according to previous results (Alarcón et al., 2016). The distance from the RCJ to the root apex was estimated to be 457 µm, which was removed before dividing the root into several zones. The root apical meristematic zone (MZ) is located in the most apical 1.5 mm of root from RCJ and the transition zone (TZ) continued to 3 mm. The fast elongation zone (EZ), where cells elongated rapidly, was assigned to 3-6 mm; and in regions far away from 6 mm we observed that cell elongation rate starts to decrease, indicating the growth terminating zone (Verbelen et al., 2006). Zones sited 12-20 mm and 20-30 mm from RCJ are differentiation zones (DF), where lateral roots initiate their development. Similar dissections of roots were made in *Arabidopsis*, where the root was dissected into five sections which are shorter than those in maize root (Cools et al., 2010). Recently, a differential gene expression along the *Arabidopsis* root has been reported, and the repressed or induced genes vary mostly in the change from the meristematic to the transition zones as well as from the transition to the elongation zone (Chaiwanon and Wang, 2015).

The limit of root meristem is determined by the exit of cell cycle and the balance between cell division and cell elongation (Perilli et al., 2012). The flow cytometry profile shows cell cycle progression along the primary root, and data showing the number of relative cells on the cell cycle phases indicate the cell activity in several root zones. Therefore, when cells exit the cell cycle, they might stop cycling. In addition, it is generally assumed that cell exit from the cell cycle takes place after mitosis, and the decision to enter a new cell cycle is made at the G1-to-S transition point in response to growth factors and various hormones (Gutierrez et al., 2002; Inzé and De Veylder, 2006; Polyn et al., 2015).

**Fig. 1.**
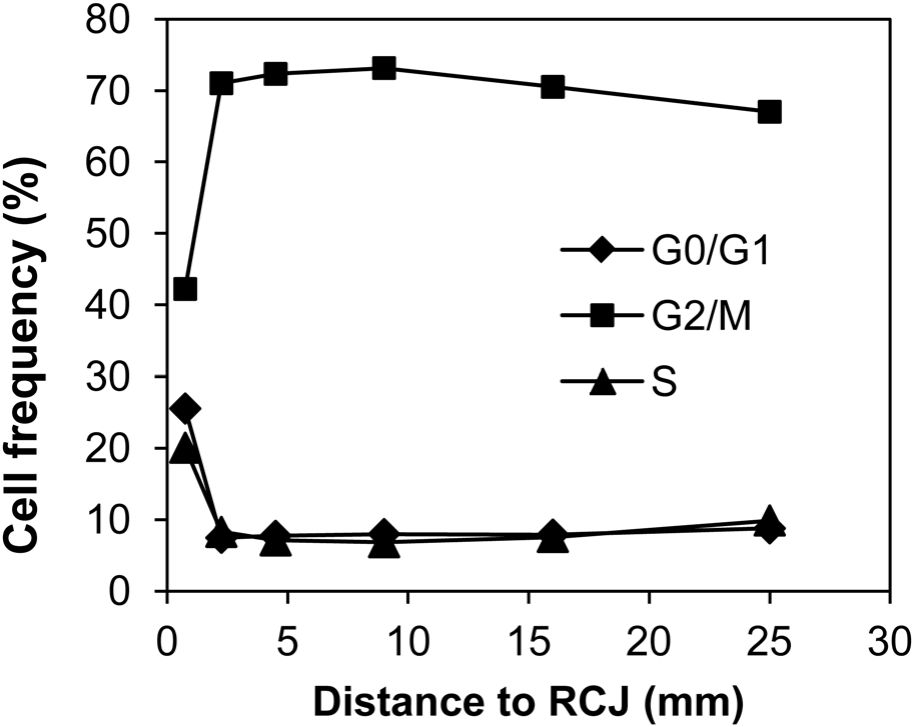
**Distribution of cell population in the several cell cycle phases along the root apex.** Roots were grown hydroponically at 30°C to reach 150-160 mm in length. Data are from an individual representative experiment using at least 10,000 cells in the estimations of percentages at different distances to the RCJ. Experiments were performed in triplicate.

**Fig. 2.**
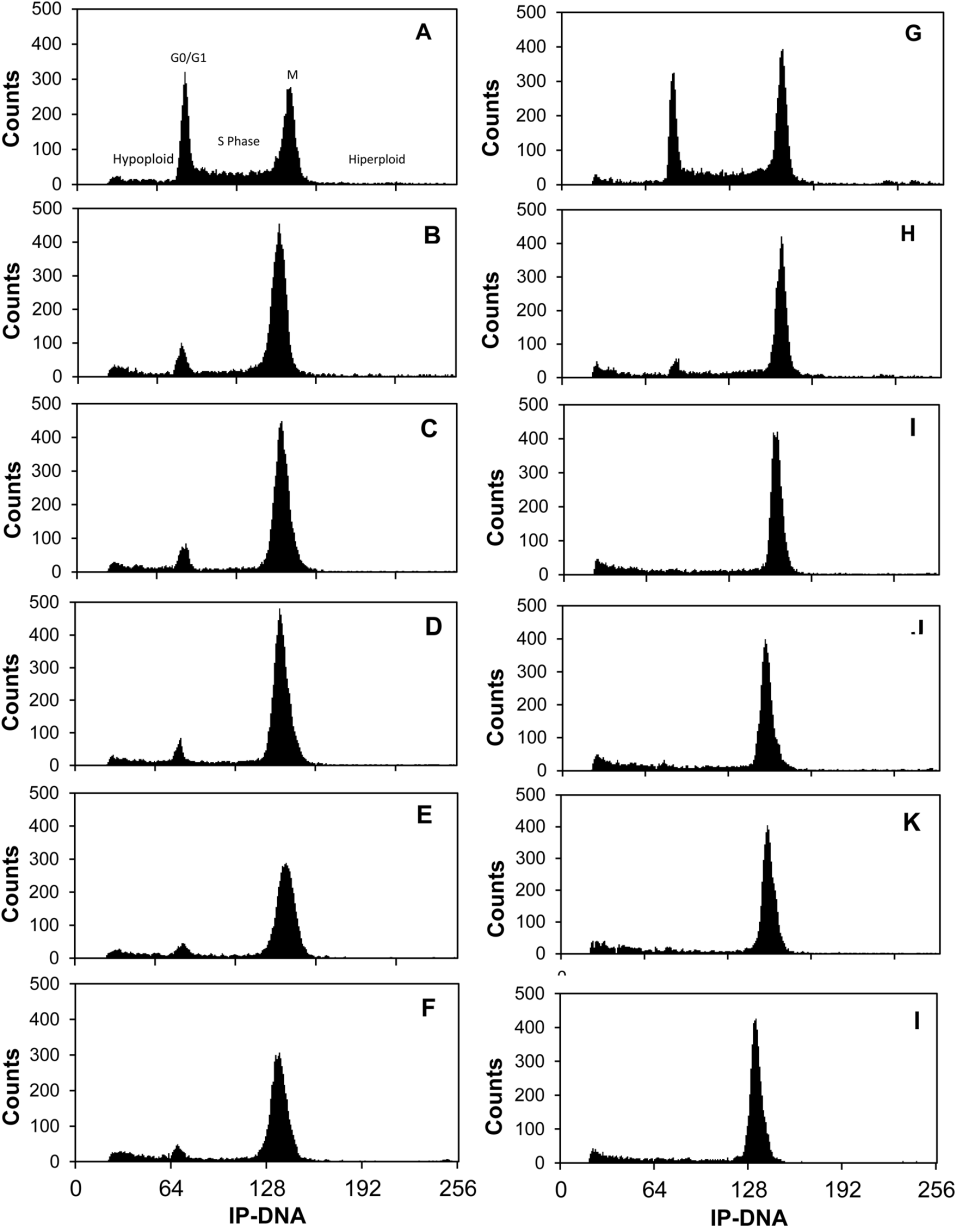
**Temperature effect on cell cycle along maize root apex.** DNA content frequency histograms representing cells from several segments of maize root apex grown at 20°C and 30°C. The left column represent histograms from roots grown at 30°C; the right column are from roots grown at 20°C. The roots were divided into several segments: 0–1.5 mm (A,G); 1.5–3 mm (B,H); 3–6 mm (C,I); 6–12 mm (D,J); 12–20 mm (E,K) and 20–30 mm (F,L). Data are from an individual representative experiment using at least 10,000 cells. Experiments were performed in triplicate.

In Fig. 1 we show that, in roots of 150-160 mm grown at 30°C, the meristem is practically restricted to MZ, the zone 0-1.5 mm from RCJ where the percentages of cell in G0/G1-, S-, and G2/M-phases were approximately 27, 23 and 39, respectively (Table 1). The relative duration for G1, S, G2 and M in *Allium* meristem has been estimated as 26.5, 44.5, 16.5 and 12.5%, respectively (Giménez-Martín et al., 1977), and these percentages are very similar in other species. Although our results reported that maize meristem has a lower percentage of cells in S-phase, the values (23%) were enough to indicate cell cycle progression (Reichheld et al., 1999). These relative proportions extraordinarily changed when TZ is analysed, in this zone. The percentages were 9, 10 and 68%, for G0/G1, S and G2/M phases, respectively. This result suggests that cells started to leave cell cycle when they reached 1.5 mm from RCJ. In addition, the percentages in EZ suggested that at 3 mm cells are out of cell cycle (Fig. 1, Table 1). In addition, the decrease in the percentage of cells in G0-G1- and S-phases was compensated with an increase in G2-M (Table 1, Fig. 2). These results clearly show that cells stop the cell cycle when they are in phase G2.

**Table 1.**
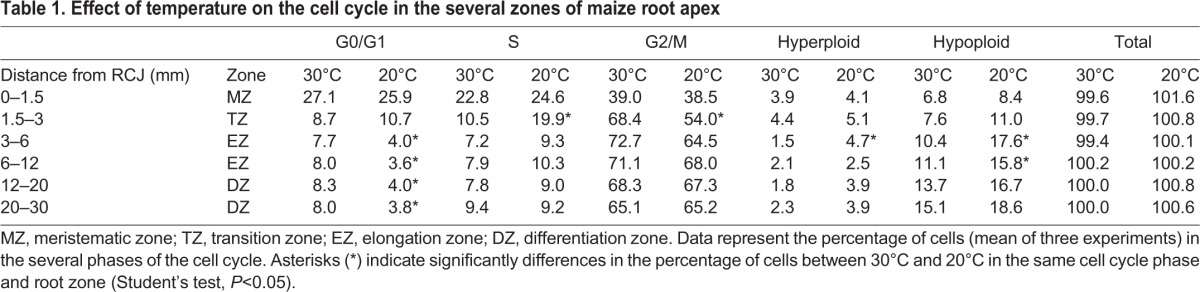
**Effect of temperature on the cell cycle in the several zones of maize root apex**

It has been reported that most of the cell types leave the meristem in G1-phase, and lately G1-to-S transition is only triggered in perycicle cells to initiate lateral root (Vanneste et al., 2007). In *Arabidopsis* pericycle, cells adjacent to protoxilem poles have been proposed to go on the cell cycle without interruption when they pass through transition, elongation and differentiation zones (Dubrovsky et al., 2008). These cells are the only ones that divide to initiate lateral root primordia. However, the rest of the cells that constitute the root do not divide. Then, epidermal and cortex cells that make up the majority of root tissues do not continue to cycle when they leave the root meristem.

In our analysis we observed a remarkable increase of cells in phase G2 in TZ and EZ. This result suggests that cells leaving meristem in phase G2 do not undergo mitosis, but they stop at this G2 phase and remain in it. We speculate that cells which exit meristem in G1- or S-phase continue the cell cycle to G2, where they stop. This is based on the observation that the decrease in percentage of cells in G0/G1 between MZ and TZ (29.7%) is compensated with an increase in G2 (28.8%) (Table 1, Fig. 2). A weak decrease in G2 percentage is achieved between the EZ and the basal end of DZ (20-30 mm from RCJ). This small diminution, together with the decrease in hyperploid nuclei, is compensated by increased hypoploid levels (Table 1).

On the other hand, it has been reported that the TZ in the maize apex root are not engaged in mitotic divisions, and in this zone postmitotic nuclei are located in a central position within the cell (Baluška et al., 2001). In addition, no mitotic figures have been observed along the root zone; lateral root primordia initiation was observed between 20-25 mm from RCJ (Alarcón et al., 2016). In *Arabidopsis*, the average distance to the earliest mitosis in the pericycle is 3194 µm and the first mitosis has been observed at 2205 µm from the root tip (Dubrovsky et al., 2001). In maize root, only at 20-30 mm from RCJ, some pericycle opposite xylem cells showed condensate cytoplasm, indicating they are re-entering the cell cycle (Alarcón et al., 2016). These differences might be caused by the greater elongation root rate in maize which grows 80-90 mm/day, whereas *Arabidopsis* elongates only 10 mm/day (Dubrovsky et al., 2001).

It has been shown using a tissue-specific quantitative microscopic analysis that some cells of cortex and epidermis were in the first endocycle (DNA contents between 4C and 8C) at their start of elongation. Moreover, nuclei of metaxylem elements in the transition zone accomplished one or two endocycles reaching 32C at their onset of rapid elongation (Baluška, 1987, 1990; Baluška and Kubica, 1984; Baluška et al., 1995). Recently, endoreduplication has been described to occur in plants before cells initiate differentiation (De Veylder et al., 2007). However, we did not observe a relevant number of nuclei with ploidy level higher than 4n. Moreover, if endoreduplication was a common process in maize root tip, we would expect hyperploidy to increase as we analysed zones further away from RCJ*.* However, hyperploidy not only did not increase in zones elongation zones, but decreased (Table 1).

It has been reported that pericycle cells remain in G1-phase until they re-enter the cell cycle (Vanneste et al., 2007). However, pericycle cells represent just a small fraction of the total amount of cells that form the root tips; epidermal and cortex cells being the most abundant types of cell in root apex. Then, if about 70% of total cells abandon meristem in G2-phase, most of the epidermal and cortex cells must be in G2 when they leave the meristem. It is assumed that epidermal and cortex cell elongation control root longitudinal growth (Alarcón et al., 2014b). Therefore, cells involved in the differentiation process that results in root elongation should be in G2 phase.

### Effect of temperature on cell cycle

In our experimental conditions, optimal temperature for maize root elongation was estimated in 30°C, and root elongation decreased by 50% when roots were grown at 20°C. The difference in the several cell cycle phases between roots elongated at 30 and 20°C is presented in Table 1.

The most relevant result was that the percentage of cells in G0/G1 at 20°C diminished by 50% compared to roots grown at 30°C in EZ and DZ. The peak corresponding to G0/G1-phases practically disappeared in flow cytometry profiles at 20°C from the segment located in TZ (3 mm away from RCJ). This indicates that cells leaving meristem in G0/G1- or S-phases continue to cycle until they reach G2-phase, where they stop. In addition, we observed that these changes in transition zone are quicker at 30°C. The changes in percentage in G0/G1 reached a stable value that did not change along the TZ, but this fact occurred in EZ when roots were grown at 20°C. In the same way, the strongest changes in S- and G2/M-phases at 30°C occurred in TZ, but they took place in the zone EZ at 20°C. It is well known that cell cycle time increased at suboptimal temperatures (Giménez-Martín et al., 1977). Therefore, cells at 30°C presented a shorter cell cycle time, as they go through the cell cycle phases more rapidly.

In summary, data reported in this work indicate that cells controlling root elongation in maize abandon meristem in G2-phase. When cells leave meristem in G1- or S-phases, they continue the cycle until they reach G2-phase, and then they stop. These results reveal the role of cell cycle on the balance between the cell proliferation and differentiation processes which occur in the meristem and the elongation zone of the root.

## MATERIAL AND METHODS

### Plant material and growth conditions

Seeds of *Zea mays* L. cv DK 626 were washed three times and soaked in distilled water with aeration at 30°C. After 24 h, the seedlings, with radicles of about 1 mm length, were placed in plastic boxes on filter paper moist with distilled water. Seed were also covered with filter paper and grown in darkness. They were kept vertically for 24 h until the roots reached a length of 30±5 mm. Discs with 10 selected seedlings of uniform root length were placed in bottles containing 1.5 l of growth medium composed of a solution of 1 mM HEPES (2-hydroxyethylpiperazine-2-ethanesulfonic acid) CaCl_2_ 1 mM and KCl 10 mM buffered growth solution, and grown at 30°C in darkness. The growth medium was aerated by an aquarium pump. After an acclimation period of 24 h, primary roots were 70-80 mm long and then roots were grown at 30°C or 20°C. Roots elongated 84.75±4.53 and 42.98±2.40 mm/day (mean±s.d.) at 30°C and 20°C, respectively. The next day, roots of 70-80 mm reached 150-160 mm (30°C) or 115-120 mm (20°C).

### Flow cytometry estimation of cell cycle progression

Primary roots grown at 30 and 20°C were dissected in several segments according to the different root zones of the root. The root cap was eliminated by removing most apical 0.5 mm and the following segments were cut: 0–1.5, 1.5–3, 3–6, 6–12 and 12–20 mm, and kept in different tubes. The segment was chopped with a razor blade for 30–60 s in a watch glass containing around 2 ml of extraction buffer [Tris-HCL 0,2 M, MgCl2-6H2O 4 mM, EDTA-Na2-2H2O 2 mM, NaCl 86 mM, Metabissulfite 10 mM, 1% PVP 10, 1% (v/v) Triton X-100 pH 7.5]. The resulting extract was passed through a 30 μm filter and centrifuged at 1500 rpm, 5 min. Then, 1 ml of staining buffer (50 µl of RNAsa 20 mg/ml, 50 µl of Propidium Iodine 0.05% and 900 µl of PBS) was added. Samples were incubated at 37°C in the dark for 30 min. Flow cytometry analysis was performed using a FC500 flow cytometer (BeckmanCoulter, Hialeah, FL, USA). At least 10,000 single nuclei (discarding doublets and aggregates) were acquired in each sample. Experiments were performed in triplicate.
